# Radiation effects on retinal layers revealed by OCT, OCT-A, and perimetry as a function of dose and time from treatment

**DOI:** 10.1038/s41598-024-53830-6

**Published:** 2024-02-09

**Authors:** Michelle R. Tamplin, Jui-Kai Wang, Elaine M. Binkley, Mona K. Garvin, Daniel E. Hyer, John M. Buatti, H. Culver Boldt, Isabella M. Grumbach, Randy H. Kardon

**Affiliations:** 1 Division of Cardiovascular Medicine, Department of Internal Medicine, University of Iowa, Iowa City, IA USA; 2 Iowa City VA Center for the Prevention and Treatment of Visual Loss, Iowa City, IA USA; 3 Division of Neuro-Ophthalmology, Department of Ophthalmology and Visual Sciences, University of Iowa, Iowa City, IA 52242 USA; 4 Department of Electrical and Computer Engineering, University of Iowa, Iowa City, IA USA; 5 Department of Radiation Oncology, University of Iowa, Iowa City, IA USA

**Keywords:** Eye cancer, Medical imaging

## Abstract

Optical coherence tomography (OCT) has become a key method for diagnosing and staging radiation retinopathy, based mainly on the presence of fluid in the central macula. A robust retinal layer segmentation method is required for identification of the specific layers involved in radiation-induced pathology in individual eyes over time, in order to determine damage driven by radiation injury to the microvessels and to the inner retinal neurons. Here, we utilized OCT, OCT-angiography, visual field testing, and patient-specific dosimetry models to analyze abnormal retinal layer thickening and thinning relative to microvessel density, visual function, radiation dose, and time from radiotherapy in a cross-sectional cohort of uveal melanoma patients treated with ^125^I-plaque brachytherapy. Within the first 24 months of radiotherapy, we show differential thickening and thinning of the two inner retinal layers, suggestive of microvessel leakage and neurodegeneration, mostly favoring thickening. Four out of 13 eyes showed decreased inner retinal capillary density associated with a corresponding normal inner retinal thickness, indicating early microvascular pathology. Two eyes showed the opposite: significant inner retinal layer thinning and normal capillary density, indicating early neuronal damage preceding a decrease in capillary density. At later time points, inner retinal thinning becomes the dominant pathology and correlates significantly with decreased vascularity, vision loss, and dose to the optic nerve. Stable multiple retinal layer segmentation provided by 3D graph-based methods aids in assessing the microvascular and neuronal response to radiation, information needed to target therapeutics for radiation retinopathy and vision loss.

## Introduction

Radiation damage to the healthy retina, or radiation retinopathy, is the primary morbidity observed after ocular radiotherapy. In patients treated with ^125^I-plaque brachytherapy for uveal melanoma, the most common intraocular cancer in adults^1^, radiation retinopathy manifests as damage to the retinal vasculature that commonly shows the first signs at 6 months to 2 years after radiotherapy^2,3^. These changes are followed by vision loss, which is often significant vision loss within 3 to 5 years of radiotherapy^2,4–6^. In recent years, optical coherence tomography (OCT) has become a widely-used method of diagnosing and observing radiation retinopathy, using presence of intra- or sub-retinal fluid or abnormal retinal thickening as the major diagnostic criteria^7,8^. Evidence of edema by OCT has been used to diagnose radiation retinopathy as many as 5 months earlier than loss of vision or detection of changes to retinal capillaries^8^, and has been used as an indication for intravitreal injections of anti-VEGF treatment. However, studies typically focus on the presence of fluid relative to the fovea^8–12^ rather than identify abnormalities of the retinal layers in addition to fluid and underlying pathology responsible for visual dysfunction. Increased thickness of the retinal nerve fiber layer (RNFL) or ganglion cell-inner plexiform layer (GCIPL) complex may indicate axonal swelling or microvessel leakage, while outer retinal thickening may indicate swelling of the photoreceptors precedent to neural atrophy and loss of visual function. Identification of the retinal layers and cell types that initially manifest change may be critical to developing preventative measures against retinal damage and eventual vision loss.

Here, we present a novel, high-resolution layer mapping approach to segment volumetric OCT scans, used to generate percentile maps that highlight regions of abnormally thickened or thinned layers of the retina. These maps are directly compared to maps of retinal capillary density by OCT-angiography (OCT-A), macular visual field sensitivity maps, and isodose maps in the same area of the macula to relate deviations in retinal layer thickness to decreased vascularity, loss of visual function, and radiation dose. For the first time, images from each modality are presented together for each patient, in chronological order of time post-radiotherapy, to determine how damage manifests in the different retinal layers over time and with respect to vascular pathology and neuronal dysfunction.

## Results

### Subject population

Of the 23 patients enrolled, the mean age was 60 years (range 28–82 years), and 35% were female (“Total”, Table 1). The average time from treatment was 31 months (range 0.25–143 months). Comorbidities included hypertension (17%), diabetes mellitus (9%), and diabetic retinopathy (9%), and 65% of patients received a clinical diagnosis of radiation retinopathy (Table 1). All patients had melanomas involving the choroid, with 22% involving the ciliary body (Table S1). The mean dose to the macula was 48 Gy; to the optic disc, 37 Gy (Table S2). A bevacizumab injection was administered to 57% of patients at the time of plaque removal, and 39% of patients received additional injections over the course of their follow-up for clinically apparent radiation retinopathy with retinal edema (Table S2). For control subjects (*n* = 104), the mean age was 48 years (range 18–82 years), and 50% were female; more detailed descriptions of their demographics were reported previously^13,14^.

**Table 1:** Subject demographics.

Features	Total no. (%), *n* = 23	Early (< 24 mo post-RT) no. (%), *n* = 13	Late (≥ 24 mo post-RT) no. (%), *n* = 10
Age (years), mean (range)	60 (28, 82)	56 (28, 81)	65 (47, 82)
Race			
Caucasian	23 (100)	13 (100)	10 (100)
Sex			
Male	15 (65)	9 (69)	6 (60)
Female	8 (35)	4 (31)	4 (40)
Medical history			
Hypertension	4 (17)	2 (15)	2 (20)
Diabetes mellitus	2 (9)	1 (8)	1 (10)
Diabetes mellitus and hypertension	1 (4)	0 (0)	1 (10)
Diabetes mellitus and diabetic retinopathy^a^	2 (9)	1 (8)	1 (10)
Radiation retinopathy^b^	15 (65)	6 (46)	9 (90)
LogMAR visual acuity, mean (range)			
Time of diagnosis	0.07 (− 0.10 to 0.40)	0.05 (− 0.04 to 0.24)	0.09 (− 0.10 to 0.40)
Time of study visit	0.13 (− 0.08 to 1.00)	0.13 (− 0.08 to 1.00)	0.14 (0–0.42)
Months post-treatment, mean (range)	31 (0.25–143)	10 (0.25–18)	58 (24–143)

^a^Diagnosis was determined by a single microaneurysm in each eye seen on digital color fundus photograph.

^b^Diagnosis was determined by physician evaluation of the central 10° of the macula seen in digital color fundus photographs.

## Summary of analysis framework

**Figure 1:**
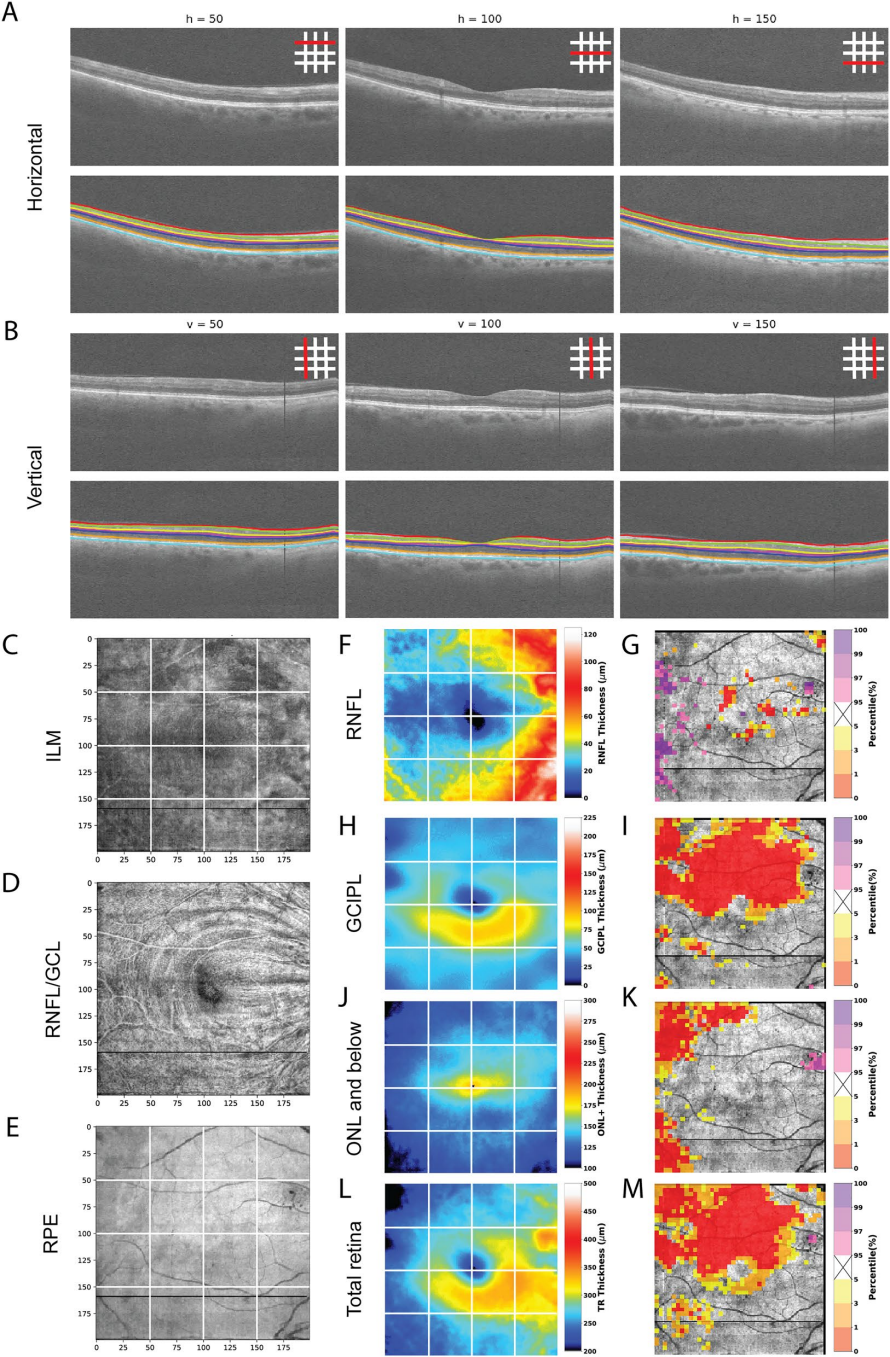
Novel OCT layer mapping methods depict severity of retinal pathology in each layer. Representative horizontal (**A**) and vertical (**B**) OCT-B scans from a patient seen at 54 months post-radiotherapy, each of the 3 horizontal and vertical B scans shown in this report are spaced every 50 B scans (approximately every 1.5 mm) in the vertical and horizontal planes. In the upper right corner of each B scan is a key showing where each B scan was sampled. The borders of each of the segmented layers are marked by colored lines: RNFL (red to yellow), GCIPL (yellow to green), ONL and below (cyan to purple), and total retina (red to purple). The inner limiting membrane (**C**), RNFL/GCIPL (**D**), and retinal pigment epithelium (**E**) surfaces were segmented to display their corresponding en face view which can show subtle changes in pixel intensity corresponding to pathological changes in each layer that may be overlooked in the color thickness maps (for example note the darker macular arcuate nerve bundle defects in the superior retina in **D**). For each layer, a thickness map (**F**, **H**, **J**, **L**) and corresponding percentile ranking map (**G**, **I**, **K**, **M**) were generated. Percentile maps depict severity of pathology compared to 104 normal control eyes and are color-coded following the schema established by Zeiss Cirrus OCT: 95th percentile and above (range light to deep purple) show abnormal layer thickening, while 5th percentile and below (range yellow to red) correspond to abnormal layer thinning.

**Figure 2:**
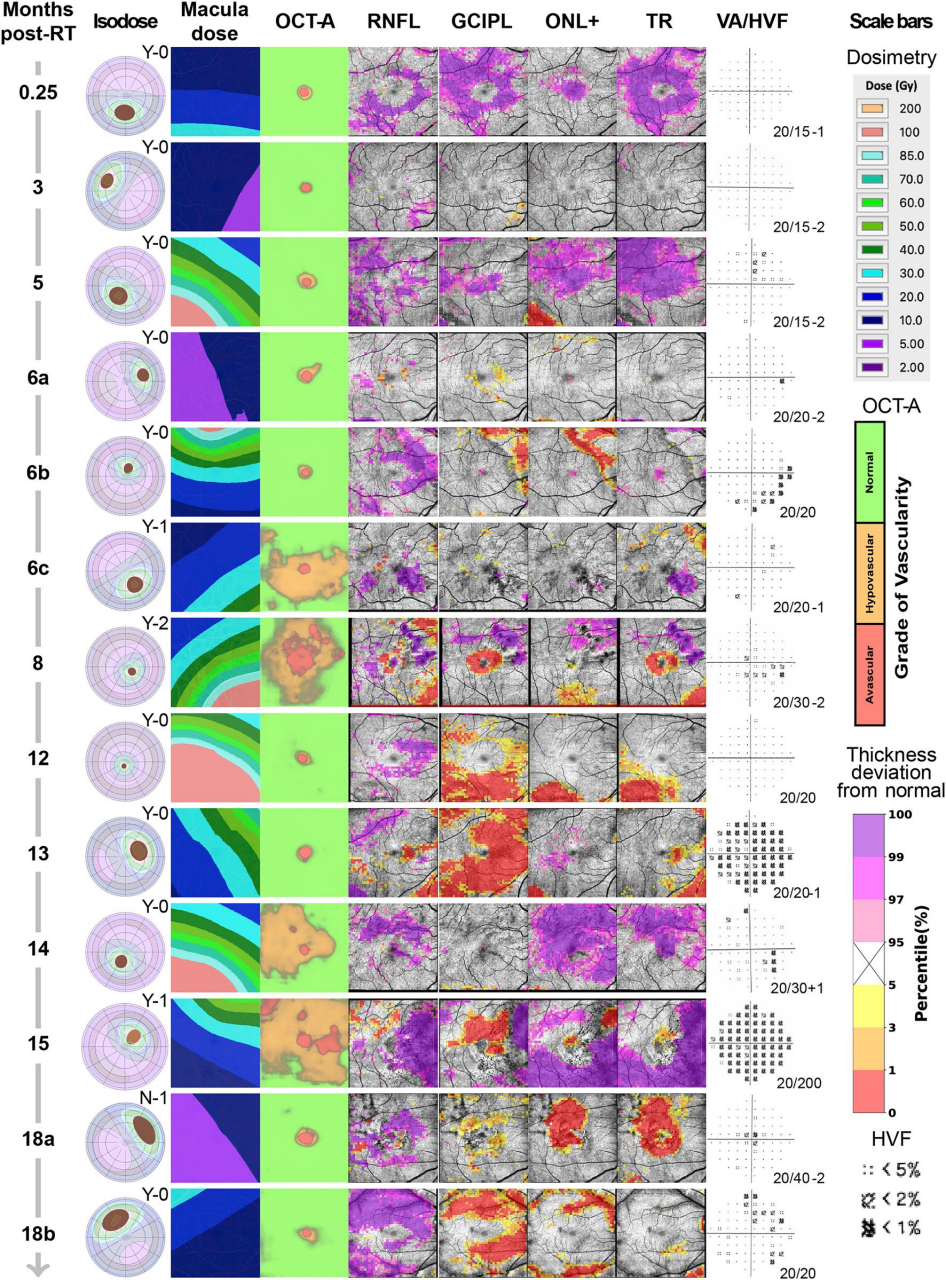
Relationship between retinal layer thickness, macular vessel density, visual field sensitivity, radiation dose, and time from radiotherapy in patients within 24 mo of ^125^I-plaque brachytherapy. Patients are sorted by increasing number of months from radiotherapy (Months post-RT), and all images are presented in right eye orientation for ease of image comparison. (Isodose) Tumor diagram with overlaid isodose lines describing size and location of the tumor relative to the macula, and radiation dose to the entire eye. The letter (Y or N) refers to whether the eye received intravitreal bevacizumab at the time of plaque removal and the associated number represents the number of subsequent bevacizumab injections. (Macula dose) 2D isodose map of radiation dose to the imaged macular area. (OCT-A) Map of graded vascularity by deep learning-trained neural network (normal, green; hypovascular, orange; avascular, red) by OCT-A. (RNFL, GCIPL, ONL+, TR) Percentile maps of each segmented layer describing abnormally thick (95th percentile and above, purple scale) and abnormally thin (5th percentile and below, yellow–red scale) regions. The actual thickness maps of each layer are provided in Fig. S1 and S2. (VA/HVF) 10–2 visual field map of visual function in the imaged macula area, with best-corrected visual acuity reported in lower-right corner of the field map. (Scale bars) Scale bars for each image readout. Note that early decrease in inner retinal capillary density by OCT-A was observed in 4 eyes at 6, 8, 14 and 15 months after radiation treatment. In these eyes there was little, if any signs of inner retinal thinning or visual field loss that spatially correlated with decrease in capillary density, indicating that microvascular pathology was preceding nerve damage. The eye at 13 months following radiation showed significant inferior thinning of the GC-IPL layer, even though no decrease in capillary density was detected or abnormal thickness in the underlying outer retinal layers.

**Figure 3:**
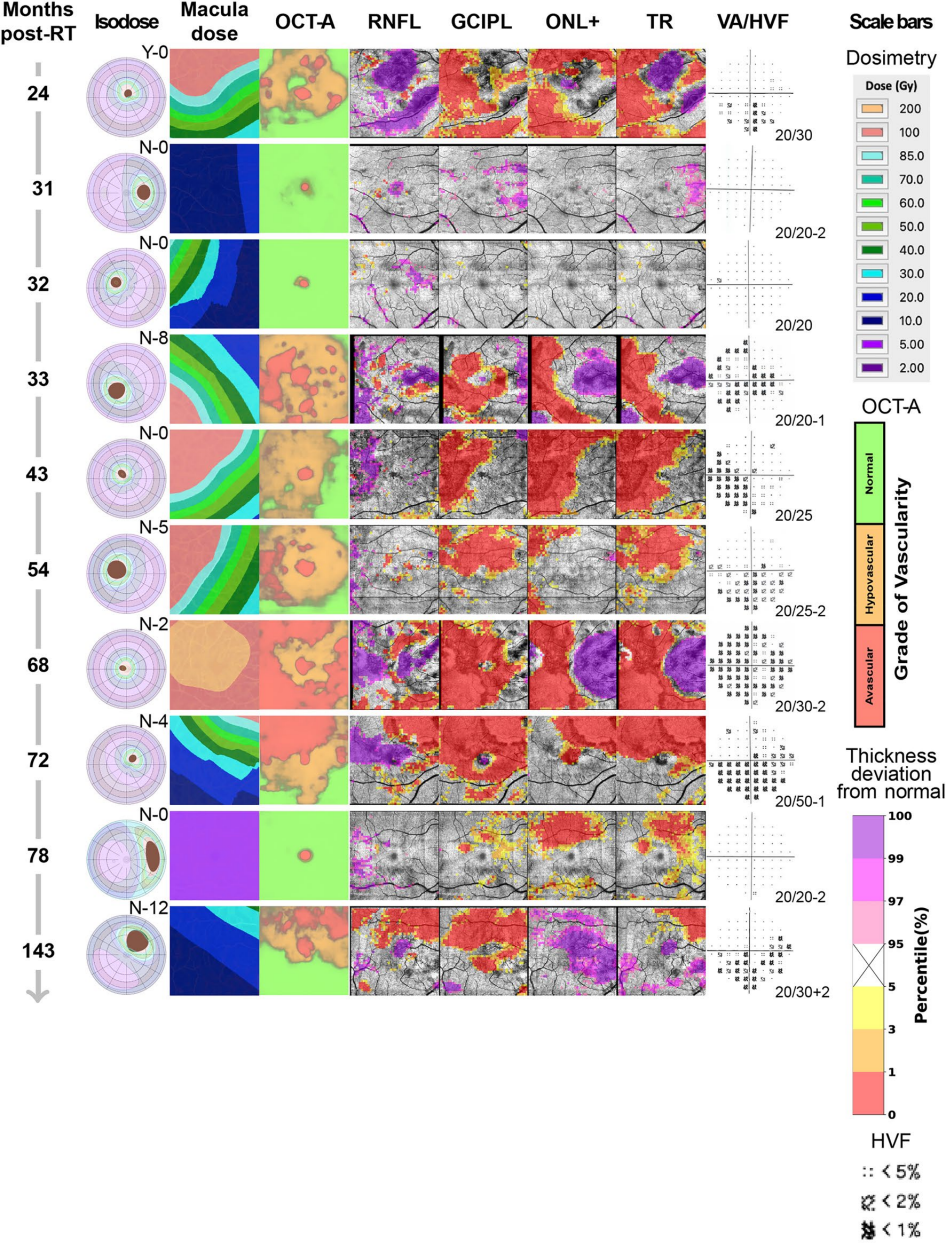
Relationship between retinal layer thickness, macular vessel density, visual field sensitivity, radiation dose, and time from radiotherapy in patients imaged 24 mo or later after ^125^I-plaque brachytherapy. Patients are sorted by increasing number of months from radiotherapy, and all images are presented in right eye orientation for ease of image comparison. (Isodose) Tumor diagram with overlaid isodose lines describing size and location of the tumor relative to the macula, and radiation dose to the entire eye. The letter (Y or N) refers to whether the eye received intravitreal bevacizumab at the time of plaque removal and the associated number represents the number of subsequent bevacizumab injections. (Macula dose) 2D isodose map of radiation dose to the imaged macular area. (OCT-A) Map of graded vascularity (normal, green; hypovascular, orange; avascular, red) by OCT-A. (RNFL, GCIPL, ONL+, TR) Percentile maps of each segmented layer describing abnormally thick (95th percentile and above, purple scale) and abnormally thin (5th percentile and below, red scale) regions. (VA/HVF) 10–2 visual field map (HVF) of visual function in the image area, with best-corrected visual acuity (VA) reported in lower-right corner of the field map. (Scale bars) Scale bars for each image readout. 2 out of 8 eyes with significant inner retinal thinning at 54 and 143 months after radiation showed a spatial pattern consistent with damage of nerve bundles at the optic nerve head (see Fig. 6). The remaining 6 eyes showed thinning in both inner and outer layers that was spatially correlated, indicating direct damage to the retina and not secondary to optic nerve damage.

For each patient, a graphical summary report was generated as a separate row, containing the following images: tumor diagram with overlaid isodose lines, isodose map of the imaged macula area, macula OCT-A map depicted as three grades of vascularity (normal, hypovascular, and avascular pixel areas)^13^, probability maps for each segmented layer (retinal nerve fiber layer (RNFL), ganglion cell plus inner plexiform layers (GCIPL), outer nuclear layer through the lower surface of the retinal pigment epithelium complex (ONL+), and total retina (TR), as shown in Fig. 1), and 10–2 visual field (HVF) probability report (Fig. 2, 3). All images were presented in right-eye orientation for direct comparison of each readout. For each retinal layer, the percentile score of each pixel in the image area occupied by abnormally thickened or abnormally thinned retina was indicated to distinguish between swelling or leakage (thickening; purple pixels in Figs. 1, 2, 3), and capillary dropout or atrophy of retinal tissue (thinning; yellow to red pixels in Figs. 1, 2, 3). Patients were further divided into early (< 24 mo post-RT, *n* = 13; Fig. 2) and late (≥ 24 mo post-RT, *n* = 10; Fig. 3) cohorts based on our previous findings of vascular pathology by 24 mo post-radiotherapy, as well as those from Collaborative Ocular Melanoma Study and other trials^2,3,13^. A detailed breakdown of demographics for these cohorts is provided in Table 1 and Tables S1–S2. No statistically significant differences in demographics or treatment characteristics were identified between the two cohorts (by unpaired t-test).

### Early RNFL and ONL+ thickening and GCIPL thinning precede late GCIPL and ONL+ thinning

**Figure 4:**
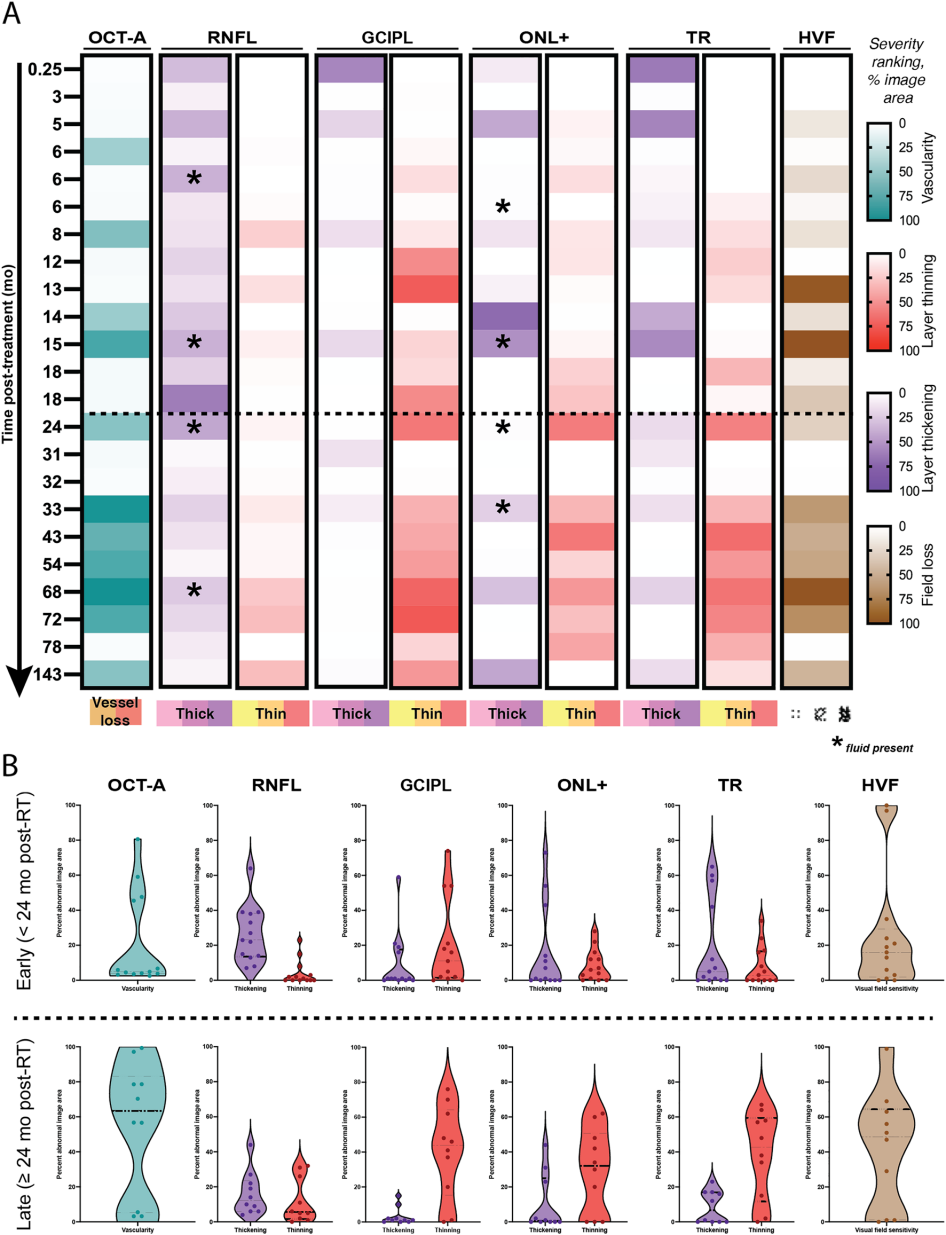
Quantitative representation of retinal vascular and neuronal readouts by time from radiotherapy. The percentage of each image categorized as abnormal based on healthy controls is ordered by months from ^125^I-plaque brachytherapy. Each row represents the treated eye of the patient seen a given time point, and columns are grouped by readout. Each retinal layer shown is displayed in two columns, for abnormal thickening (purple) and thinning (red). Layers with abnormal thickening due to intraretinal fluid are indicated with an asterisk. (**B**) Violin plots illustrate the distribution of pathology by outcome measure across all subjects, as well as the shift in prevalence across the early and late cohorts. Each point corresponds to the percentage value for each patient reported in (**A**). Dashed horizontal line in (**A**, **B**) indicates division between early (< 24 mo post-RT) and late (≥ 24 mo post-RT) cohorts.

**Figure 5:**
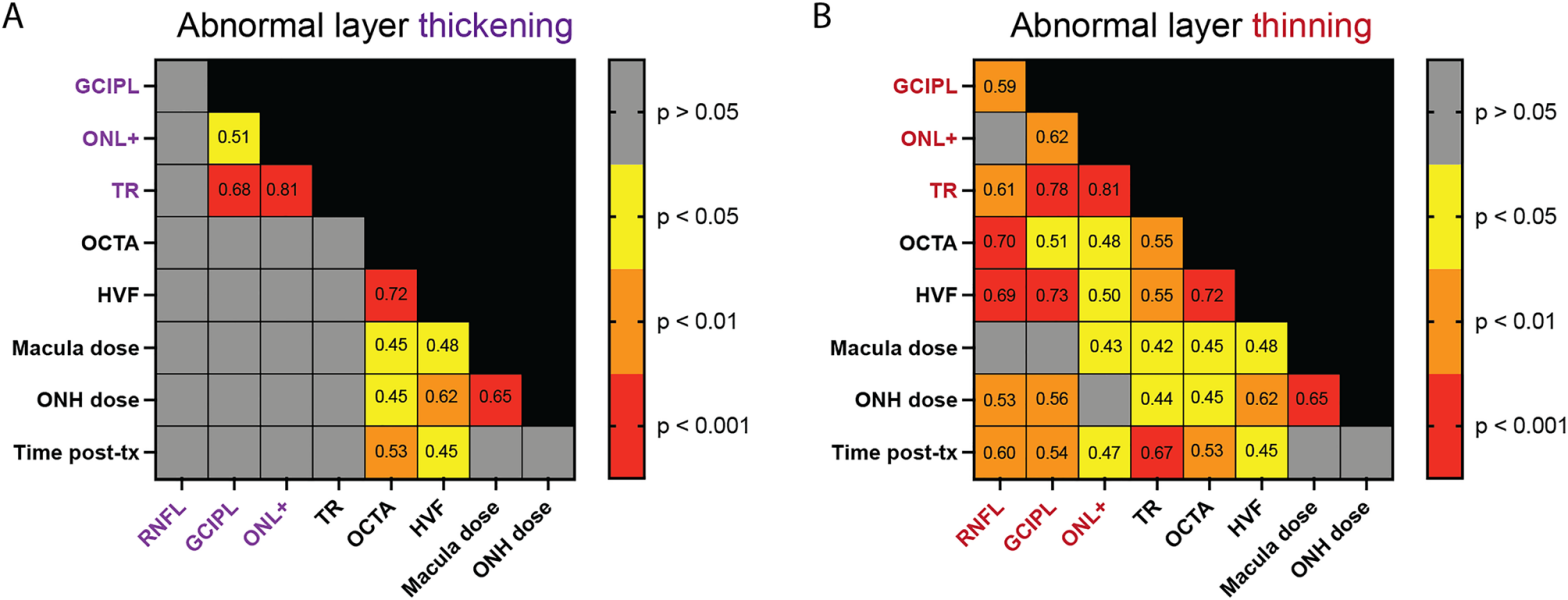
Spearman correlation matrices of readouts. Interrelationships between outcome measures are divided by abnormal layer thickening (**A**) and abnormal layer thinning (**B**) to distinguish between the two etiologies. Spearman *r* values are reported for each statistically significant relationship (*p* < 0.05) and color-coded by *p* level.

The most severe retinal thickening was observed in the RNFL and the complex layer between the outer nuclear layer plus the retinal pigment epithelium (denoted as ONL +) at early time points (< 24 mo post-RT; Figs. 2, 4; see > 95th percentile purple pixel areas in eyes at 0.25, 5, 6b, 14, 15 and 18b months). In the RNFL, a wide distribution of abnormal thickening was observed (Fig. 4B), with more than 20% of the image area abnormally thick in 8 out of 13 patients. When there was abnormal thickening of the ONL+, it was either extensive (> 40% of the image area, in 3 out of 13 patients), or focal (7–14% in 3 out of 13 patients; Figs. 2, 4). Thickening of the RNFL and ONL+ layers was still observed at late time points (≥ 24 mo post-RT), but to a much lesser degree (Figs. 3, 4). Abnormal thickening directly attributable to fluid in either the RNFL or the ONL+ was detected in 3/13 patients at early time points, and 3/10 patients seen at late time points (indicated by asterisk in Fig. 4A), indicating that not all layer thickening was due to extracellular fluid. No significant correlations between abnormal thickening, radiation dose, or time from radiotherapy were observed (Fig. 5A).

Abnormal retinal thinning was noted as early as 6 mo post-RT in the GCIPL and ONL+, with the most severe early thinning noted in the GCIPL (> 15% of image area in 6/13 patients, *vs* 3/13 for the ONL+; Figs. 2, 4). Thinning in these layers worsened significantly with time from radiotherapy (Fig. 5B), with thinning of the GCIPL only slightly more severe than in the ONL+ (Figs. 3, 4). This appeared to correspond with higher radiation doses (Figs. 2, 4). Interestingly, thinning of the GCIPL and RNFL correlated significantly (*p* < 0.01) with dose to the ONH, but not dose to the macula. Thinning of the ONL+ correlated weakly only to dose to the macula (*p* < 0.05, Fig. 5B). In Fig. 2, eyes at 6c, 8, 14, and 15 months post-RT showed significant decrease in inner retinal capillary density, yet thickness of the corresponding layers was not significantly affected, pointing towards early effects of radiation to the inner retinal capillaries before neuronal loss occurred. In contrast, eyes at 13 and 18b months showed significant thinning of the inner retinal layers while capillary density in those layers was still normal, indicating more direct damage to inner retinal neurons independent of capillary density.

### Inner retinal layer thinning correlates with decreased vascularity and visual function

The relationships between vascular pathology, loss of visual function, and retinal layer thickening or thinning were substantiated using Spearman correlation matrices (Fig. 5). Additional correlations for the early and late time groups were calculated (Fig. S4) to detect potential relationships between measures unique to the early and late post-radiation timeframes. Significant correlations with vascular pathology and loss of visual function were observed for abnormal layer thinning, but not thickening. Strong correlations (*r *≈ 0.7, *p* < 0.001) were observed between loss of visual function and thinning of the GCIPL and RNFL (Fig. 5B), driven by patients seen at later time points (Fig. S4D). Decreased inner retinal capillary density correlated more strongly with RNFL thinning (*r* = 0.7, *p* < 0.001) than thinning of the GCIPL (*r *≈ 0.5, *p* < 0.05, Fig. 5B). Very weak correlations (*r *≈ 0.5, *p* < 0.05) were observed between decreased inner retinal capillary density, loss of visual function, and thinning of the ONL+segment (Fig. 5B).

## Discussion

In this study, we utilized methods of retinal layer segmentation and associated percentile color maps to independently relate thickening and thinning of the RNFL, GCIPL, and outer retinal (ONL+) layers to inner retinal vascularity, visual function, radiation dose, and time from ^125^I-plaque brachytherapy for uveal melanoma. By presenting maps of vessel density, layer thickness, and visual function standardized to control populations, significant changes in each readout could be visually identified and interpreted within the context of the other measurements, radiation dose, and time from radiotherapy. Using this method, we identified significant thinning of the GCIPL starting as early as 6 mo post-radiotherapy, while thickening of all other layers was common but most pervasive in the RNFL and outer retina, especially within the first 24 months of radiation treatment. Detailed examination of the OCT B scans in eyes with layer thickening revealed cases of thickening due to intraretinal fluid. However, there were some eyes showing retinal thickening greater than the 95th percentile of normal that showed no evidence of fluid on OCT analysis. This could be due to subclinical vascular leakage, as some of these eyes did show fluid on OCT months later. Fluorescein angiography may be capable of revealing subclinical leakage at such an early stage. However, fluorescein angiography was not part of our clinical protocol, so this question could not be addressed by this study. Other causes of layer thickening could be due to intracellular edema, especially in the RNFL which may become thickened due to axoplasmic flow stasis or focal ischemia resulting in cotton wool spots.

**Figure 6:**
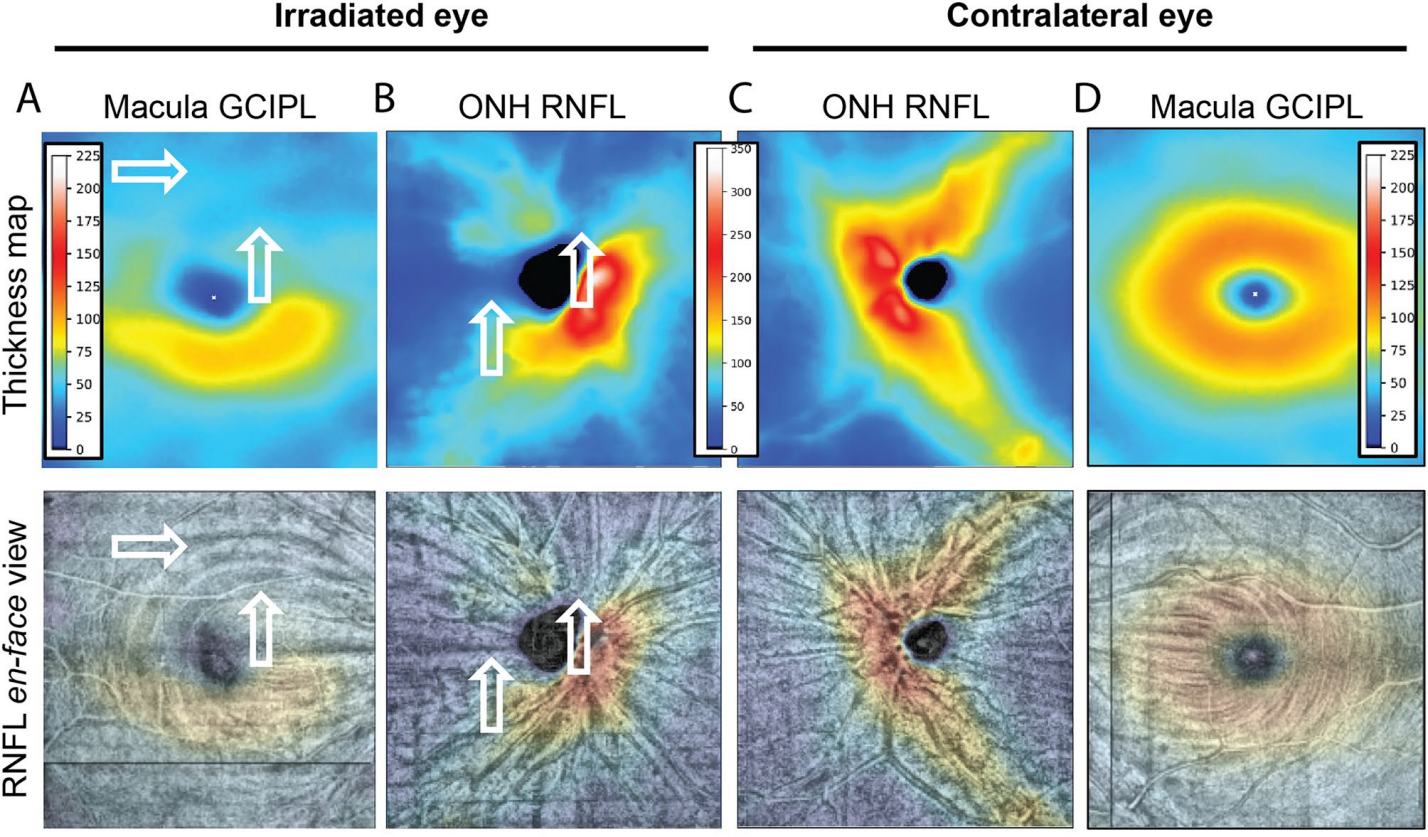
Segmentation of macula and ONH scans and en face view reveals involvement of optic nerve and associated nerve bundle damage in macular pathology. Same eye and time point as shown in Fig. 1, but ONH, thickness plots and en face views added to reveal that visible thinning of the GCIPL layer of the macula in the irradiated (**A**) compared to the contralateral eye (**D**) is accompanied by arcuate nerve bundle defects in both the macula and ONH-centered OCTA scan originating from the optic nerve head of the irradiated (**B**) but not contralateral (**C**) eye of a patient seen at 54 mo post-radiotherapy. The affected superior bundles appear as dark pixels in thin RFNL arcuate bundles and are indicated by white block arrows in the thickness maps (top) and RNFL en-face views (bottom). Color scale bars are in μm.

We showed that radiation dose to the optic nerve head, but not dose to the macula, correlates to thinning of the inner retina, and that visual field defect correlates more strongly to dose to the optic nerve head than to the macula. Although we found examples of thinning of the inner and outer retina that spatially correlated, indicative of direct damage to retina, the significant correlation of inner retinal thinning with optic nerve radiation dose may indicate a greater susceptibility of the optic nerve and inner retina to radiation. For example, it has been shown in experimental models of ionizing radiation that mitochondria are susceptible to damage involving their DNA, free-radical formation and oxidative metabolism^15–17^. The inner retina and outer retina have a significant number of mitochondria, but the density is particularly high at the optic nerve head. Mechanisms of radiation susceptibility of neural tissue are thought to involve the microvascular endothelial cells, free radical production in neuronal mitochondria, and glial cell damage^18^. After 24 months following radiation, we identified significant correlations between thinning of the inner retina, decreased vascularity, and loss of visual function. This could be explained by the damaging effects of radiation on the optic nerve head, which would account for the retrograde thinning of the axons in the RNFL and cell bodies and dendrites of the retinal ganglion cells in the GCIPL (Fig. 6), as well as an obligatory loss of inner retinal vascularity due neuron loss in the inner retinal layers due to neurovascular coupling^19^. Of note, for patients with concurrent inner retinal thinning, decreased vascularity, and loss of visual function, the mean dose to the optic nerve head was 47 Gy (range 22–73 Gy), consistent with reported thresholds of 30–60 Gy for development of optic neuropathy^20^.

Thinning of the GCIPL prior to resolution of RNFL swelling was detected in 9 patients seen at early time points (Fig. 2); all but one patient (seen at 3 mo) has visual field defects in the same areas. These findings mirror OCT studies of patients with nonarteritic anterior ischemic optic neuropathy^21^, namely that GCIPL thinning correlates better with visual field loss than thickening of the RNFL, indicating GCIPL thinning may be a robust biomarker for early structural injury after optic nerve head exposure to radiation. We further showed dose to the optic nerve, but not to the macula, correlates with GCIPL and RNFL thinning (Fig. 5), and that at later time points, GCIPL thinning, visual field loss, and decreased vascularity correlated significantly (Fig. 3, S4D). These correlations may represent a pattern of retrograde inner retinal damage, similar to what has been observed in patients diagnosed with optic neuropathy in diseases like multiple sclerosis and glaucoma^22,23^. Applying the segmentation methods presented here to a serial, longitudinal imaging study starting before radiotherapy could reveal whether this nerve damage is due to direct nerve injury or a consequence of vascular injury and ischemia. The multimodal approach utilizing OCT/OCT-A segmentation, visual field sensitivity with associated probability maps, and dosimetry is particularly well-suited towards assessment of individual patients over time. This approach will help determine which retinal layers and capillary beds are being affected over time leading to the development different therapeutic strategies aimed at specific targets in the retina and optic nerve that could constitute a form of precision treatment.

Because this was a cross-sectional study, direct causal relationships between changes in retinal thickness, microvessel loss, and visual field defects could not be definitively established; however, these relationships may be identified by applying the presented analysis framework to a cohort of patients imaged serially starting pre-radiotherapy. We are currently collecting such longitudinal data. Additionally, measurement of axial eye length, which was not available in this study, would help confirm that retinal layer thinning is caused by radiation damage and not due to intrinsic anatomical differences resulting from longer axial eye length. For patients for whom refractions were available, only 4 patients were highly myopic (sphere < − 5.00); their images did not show retinal thinning that would be considered outlier data. Furthermore, no significant thinning was observed in a separate analysis of the fellow, nonirradiated eyes, suggesting potential retinal thinning due to longer axial eye lengths was not a significant factor in this cohort. Moreover, the radiation doses used here were mean values over the entire image area; more granular analyses where both the total dose and rate of dose delivery are calculated specifically for areas of pathology or dysfunction will provide more insight into outcome-specific dose responses, as described in our previous case report^24^. Finally, our analyses here focused only on the macula and inner retinal capillaries, as this is the often the primary site of early vision loss in radiation retinopathy; however our findings suggest future studies should also include analysis of the choriocapillaris supplying the outer retina, outer retinal layer thickness and retinal layer thickness outside of the macula, including the peripapillary retina and optic nerve head.

## Methods

### Study subjects

In this single-center study, 23 patients with posterior uveal melanoma treated with ^125^I-plaque brachytherapy were consecutively enrolled from the Retina and Vitreous Clinic at the University of Iowa Hospitals and Clinics as part of a previously-reported study^13^. This study adhered to the tenets of the Declaration of Helsinki and was approved by the University of Iowa Institutional Review Board. Written informed consent was provided by all participants when enrolling in the study. Patients were imaged once at a follow-up visit ranging from 2 weeks to 12 years post-plaque removal. Detailed subject demographics, treatment characteristics, and exclusion criteria were reported previously^13^. Importantly, two of the 25 previously-reported patients were excluded here due to glaucoma-suspect features on their OCT thickness maps, to avoid confounding the analysis of radiation-specific pathology and dysfunction. For control subjects, 52 right and 52 left eyes were randomly selected from 104 normal subjects recruited at the University of Iowa. Control subjects were pre-screened by OCT (Cirrus HD-OCT 5000, Carl Zeiss Meditec, Inc. Dublin, CA) for retinal nerve fiber and ganglion cell layer thickness ranked within the 5th–95th percentile range of the device’s age-matched normative database, as described previously^13^. All control subjects were further evaluated by an neuro-ophthalmologist (RHK) to confirm these results. For data reporting and image comparison, all eyes were flipped to OD orientation.

### Image analysis

The methodology for acquisition and analysis of OCT(-A) scans, 10–2 visual field maps, and visual acuity, as well as generation of isodose maps, was conducted as previously described^13^. Briefly, for OCT(-A), a fovea-centered OCT image volume pair (304 × 304 × 640 voxels covering 6 × 6 × 2 mm^3^) was acquired using Optovue XR Avanti System (Optovue, Inc.). Graded vascularity (avascular, hypovascular, and normal vascularity) of the superficial capillary plexus was determined using our previously reported deep-learning approach^13,25^. In particular, vascularity was estimated using a modified version of the traditional U-Net^26^ with dilated convolutions, batch normalization, and a three-channel input, concatenating the OCT-A *en face* image and two directional maps. The network outputs a three-channel probability map that corresponds to each of the vascularity labels, assigned based on the label with the highest probability for each pixel location. Visual field testing was performed by Swedish Interactive Threshold Algorithm standard automated perimetry with a Humphrey Field Analyzer (Humphrey Field Analyzer Model 750i, Carl Zeiss Meditec, Inc., Dublin, CA) using the 10–2 protocol. Isodose maps were constructed for the macula and optic nerve head using the ocular brachytherapy planning software Plaque Simulator (v.6.6, EyePhysics LLC, Los Alamitos, CA), as previously described^13,14^.

For segmentation of the retinal layers (Fig. 1A), a 3D graph-based approach^27^ was applied to all of the OCT volume scans to identify the locations of the retinal nerve fiber layer (RNFL), ganglion cell plus inner plexiform layers (GCIPL), outer nuclear layer through the lower surface of the retinal pigment epithelium complex (ONL+), and total retina (TR). For each OCT volume, the fovea location was automatically identified and passed by quality control. For each layer, a thickness map (Fig. 1B, D, F, H) and corresponding probability map (Fig. 1C, E, G, I), calculated by comparing each 5 × 5 pixel region of the thickness map with the 104 control eyes, were generated. The probability (or, percentile) maps were superimposed over each patient’s *en-face* image of the retinal pigment epithelium complex, and color-coded following methods established by Zeiss Cirrus OCT measurement printouts. The 95th percentile and above (light to deep purple) correspond to abnormal layer thickening, while the 5th percentile and below (yellow to red) correspond to abnormal thinning compared to the control subjects. No adjustments were made to layer thickness to account for biological sex^28^, as no significant differences were detected between males and females for any layer (by two sided *t* test). Layer thickness maps along the A-scan direction generated for each patient, before standardization to the control population, are provided in Fig. S1–S2.

### Quantitative analyses

For each outcome measure, data were presented as the percent of the image area ranked as abnormal. For abnormal retinal thickening, this was the percentage of pixels in the 95th percentile and above; for abnormal thinning, the 5th percentile and below. For vascularity, the percentage of the image area ranked as hypovascular (orange, Figs. 2, 3) or avascular (red, Figs. 2, 3) was used. For visual field sensitivity, the percentage of test points ranked in the 5th percentile or below was used. To quantitatively assess the relationship between each measure and time from radiotherapy, the percentage values for each patient were reported as a heat map versus time from radiotherapy (Fig. 4A); to depict changes in distribution of pathology across early and late time groups, violin plots were generated from the same data (Fig. 4B). The percentage values used to generate Fig. 4 are provided in Fig. S3.

To identify relationships between abnormal thickening or thinning of the retinal layers with each outcome measure, Spearman correlation matrices (two-tailed, 99% confidence interval) were generated using GraphPad Prism (v.9.4.1 for macOS, GraphPad Software, San Diego, CA). For each layer segment, the percentage of abnormally thick (Fig. 5A) or thin (Fig. 5B) pixels was used for the correlation.

### Supplementary Information

Supplementary Information.

## Data Availability

The datasets generated during and/or analyzed during the current study are available from the corresponding author on reasonable request.
