# Supplementary material for: Radiation effects on retinal layers revealed by OCT, OCT-A, and perimetry as a function of dose and time from treatment
Source: Sci Rep. 2024 Feb 9;14:3380. doi: 10.1038/s41598-024-53830-6 (PMC10858219; doi:10.1038/s41598-024-53830-6)
Supplement: Supplementary file 1 — Supplementary Information. [file 41598_2024_53830_MOESM1_ESM.pdf]

Supplementary Table 1: Pre-radiotherapy tumor features.

Features	Total<br>no. (%), *n*=23	Early<br>no. (%), *n*=13	Late<br>no. (%), *n*=10
Involved uveal tissues			
Choroid[1]	18 (78)	9 (69)	9 (90)
Ciliochoroidal[2]	5 (22)	4 (31)	1 (10)
Tumor quadrantic location[3]			
Superior	4 (17)	2 (15)	2 (20)
Superiotemporal	4 (17)	3 (23)	1 (10)
Superionasal	0 (0)	0 (0)	0 (0)
Temporal	0 (0)	0 (0)	0 (0)
Inferior	3 (13)	3 (23)	0 (0)
Inferiotemporal	1 (4)	1 (8)	0 (0)
Inferionasal	1 (4)	1 (8)	0 (0)
Nasal	4 (17)	2 (15)	2 (20)
Peripheral macula	6 (26)	1 (8)	5 (50)
Largest basal diameter (mm), mean (range)	11.0 (4.3-17.0)	11.3 (4.3-15.0)	10.6 (6.0-17.0)
Thickness (mm), mean (range)	2.7 (1.2-4.4)	2.8 (1.2-4.4)	2.6 (1.7-3.7)
Distance to the optic disc (mm), mean (range)	5.2 (0-17.5)	5.7 (0-17.5)	4.5 (0-11.5)
Distance to the foveola (mm), mean (range)	5.9 (0-15.5)	7.0 (1.5-15.0)	4.4 (0-15.5)

[1] *Includes choroidal melanomas not involving the ciliary body or the peripapillary region.*

[2] *Includes choroidal melanomas involving the ciliary body.*

[3] *Location with respect to the optic disc.*

Supplementary Table 2: Radiotherapy and adjuvant treatment features.

**Features**	**Total** value, *n*=23	**Early** value, *n*=13	**Late** value, *n*=10
Plaque shape, no. (%)			
Round	17 (74)	10 (77)	7 (70)
Notched	6 (26)	3 (23)	3 (30)
Plaque size (mm), mean (range)	17 (14-22)	18 (14-20)	17 (14-22)
Hours of radiation, mean (range)	111.3 (94.7-122.2)	111.3 (94.7-122.2)	111.3 (96.0-121.5)
Radiation dose (Gy), mean (range)			
Prescription dose[1]	86.0 (82.0-107.6)	86.7 (82.8-107.6)	85.0 (82.0-88.9)
Tumor apex[2]	107.0 (79.4-133.1)	103.2 (79.4-118.6)	111.9 (99.9-133.1)
Optic disc[3]	36.5 (8.6-81.5)	34.9 (8.6-81.5)	38.6 (10.1-73.0)
Foveola[4]	49.4 (9.2-163.2)	33.1 (9.7-82.1)	70.6 (9.2-163.2)
Macula[5]	48.3 (7.4-178.9)	38.3 (10.4-103.8)	61.3 (7.4-178.9)
Bevacizumab injections			
At plaque removal, no. (%)	13 (57)	12 (92)	1 (10)
Over follow-up, no. (%)	9 (39)	4 (31)	5 (50)
No. of bevacizumab injections, mean (range)	4 (1-12)	1 (1-2)	6 (2-12)

[1] *Dose prescribed to the base of the tumor.*
[2] *Dose calculated at a point 1 mm past the prescription point, calculated for treatment planning purposes.*
[3] *Dose calculated to 4.5 x 4.5 mm^2 area centered on optic disc, using custom dosimetry methods.*
[4] *Dose calculated at center of the fovea, calculated for treatment planning purposes.*
[5] *Dose calculated to the imaged 6 x 6 mm^2 area of macula using custom dosimetry methods.*

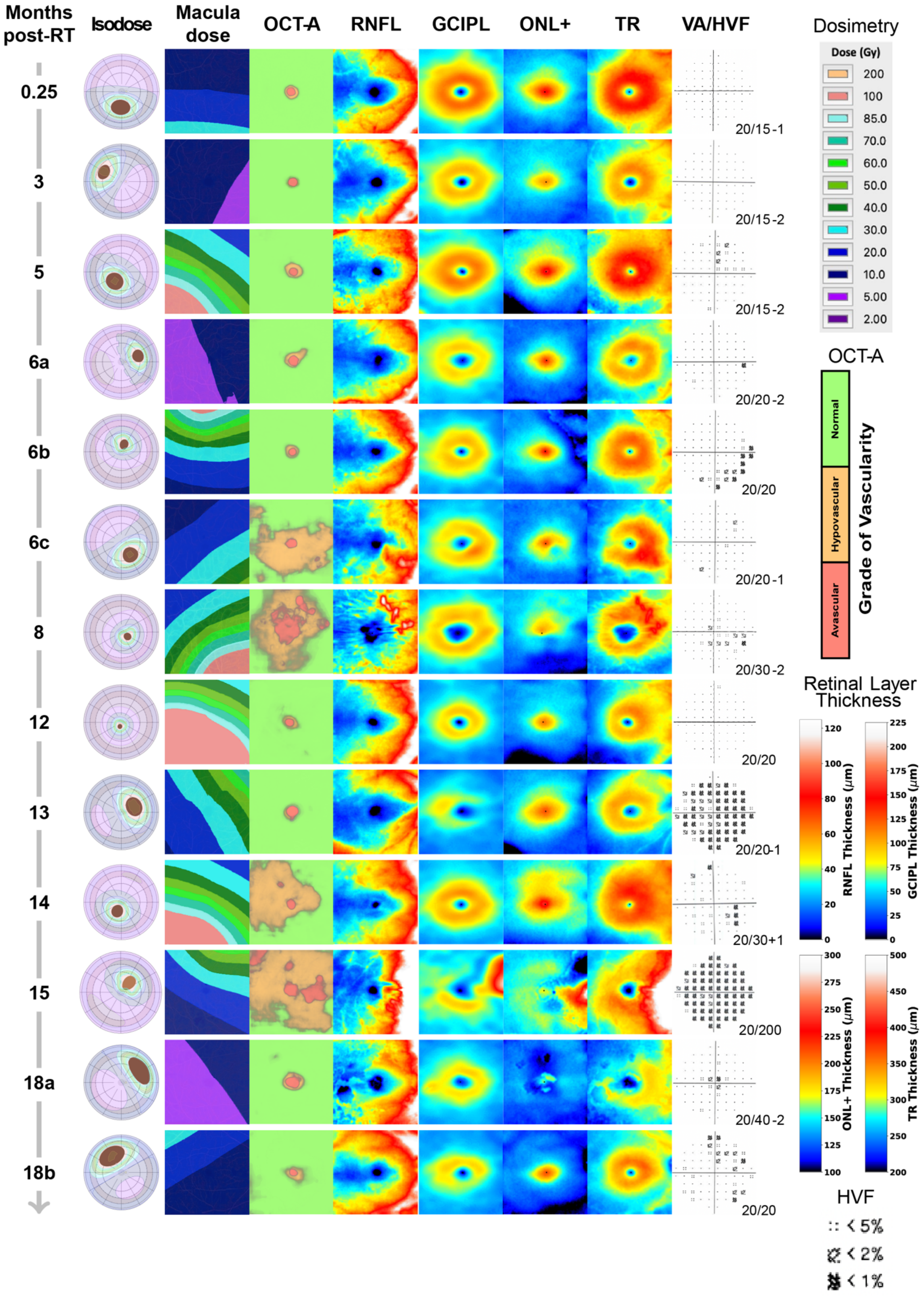

Months post-RT
Isodose
Macula dose
OCT-A
RNFL
GCIPL
ONL+
TR
VA/HVF
0.25
3
5
6a
6b
6c
8
12
13
14
15
18a
18b
20/15-1
20/15-2
20/15-2
20/20-2
20/20
20/20-1
20/30-2
20/20
20/20-1
20/30+1
20/200
20/40-2
20/20
Dosimetry
Dose (Gy)
200
100
85.0
70.0
60.0
50.0
40.0
30.0
20.0
10.0
5.00
2.00
OCT-A
Normal
Hypovascular
Avascular
Grade of Vascularity
Retinal Layer Thickness
RNFL Thickness (μm)
GCIPL Thickness (μm)
ONL+ Thickness (μm)
TR Thickness (μm)
HVF
< 5%
< 2%
< 1%

Supplementary Figure 1 (companion to manuscript Figure 2): Relationship between retinal layer thickness, macular vessel density, macular vessel density, visual field sensitivity, and time from radiotherapy in patients imaged within the first 24 mo of ^{125}I-plaque brachytherapy. From left to right: retina diagram showing tumor size and location, and global dose distribution; radiation dose distribution to the central macula; categorical vessel density by OCT-A; layer thickness for the RNFL, GCIPL, ONL and below, and total retinal thickness; visual field report and visual acuity. Except for the retinal diagram, all outcome measures are for a 6 x 6 mm^2 area of the macula centered on the foveal avascular zone. All images were flipped to OD orientation for ease of image review.

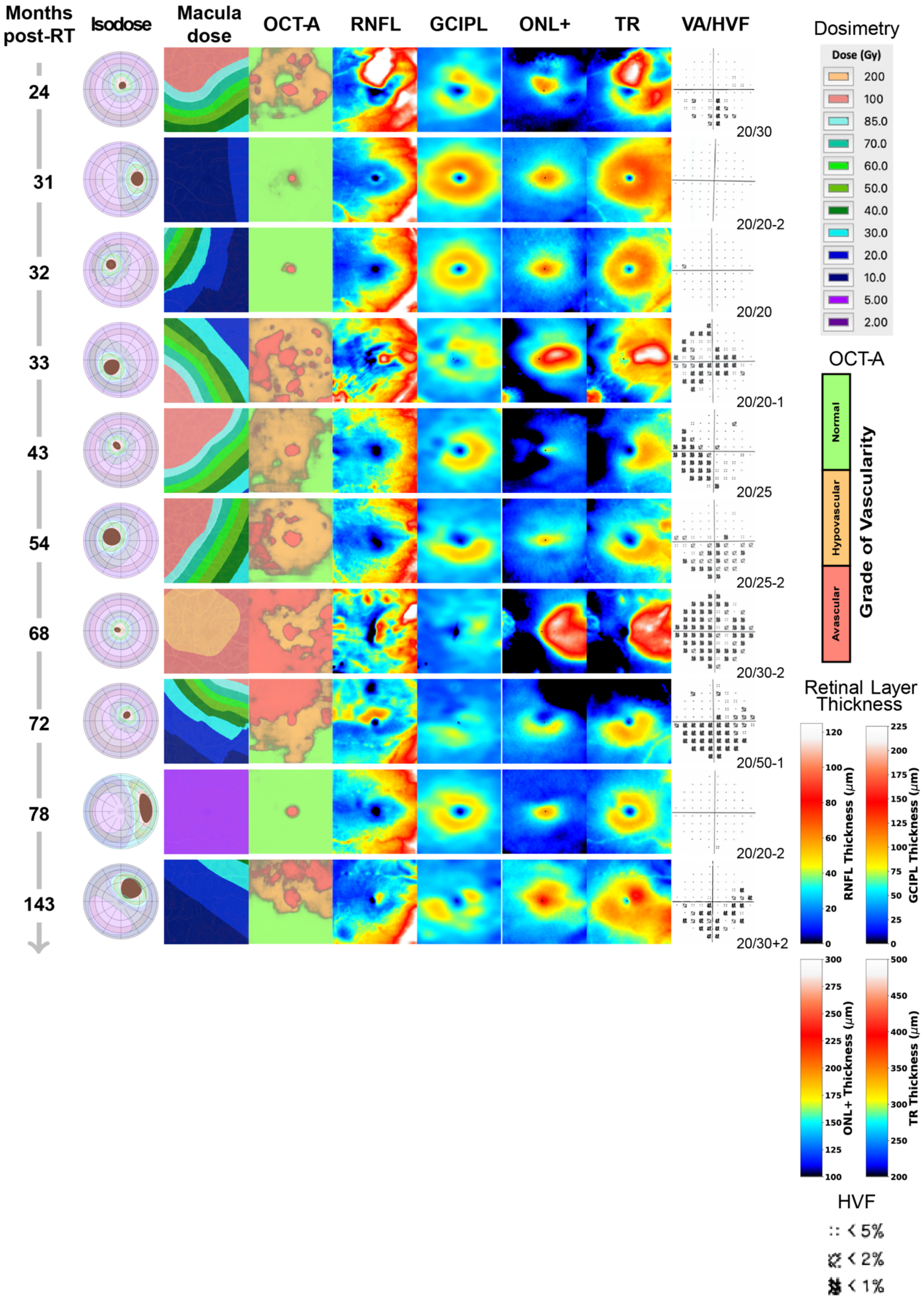

Months post-RT
Isodose
Macula dose
OCT-A
RNFL
GCIPL
ONL+
TR
VA/HVF
24
31
32
33
43
54
68
72
78
143
20/30
20/20-2
20/20
20/20-1
20/25
20/25-2
20/30-2
20/50-1
20/20-2
20/30+2
Dosimetry
Dose (Gy)
200
100
85.0
70.0
60.0
50.0
40.0
30.0
20.0
10.0
5.00
2.00
OCT-A
Normal
Hypovascular
Avascular
Grade of Vascularity
Retinal Layer Thickness
RNFL Thickness (μm)
GCIPL Thickness (μm)
ONL+ Thickness (μm)
TR Thickness (μm)
HVF
< 5%
< 2%
< 1%

Supplementary Figure 2 (companion to manuscript Figure 3): Relationship between retinal layer thickness, macular vessel density, macular vessel density, visual field sensitivity, and time from radiotherapy in patients imaged at 24 mo or later after ^{125}I-plaque brachytherapy. From left to right: retina diagram showing tumor size and location, and global dose distribution; radiation dose distribution to the central macula; categorical vessel density by OCT-A; layer thickness for the RNFL, GCIPL, ONL and below, and total retinal thickness; visual field report and visual acuity. Except for the retinal diagram, all outcome measures are for a 6 x 6 mm^2 area of the macula centered on the foveal avascular zone. All images were flipped to OD orientation for ease of image review.

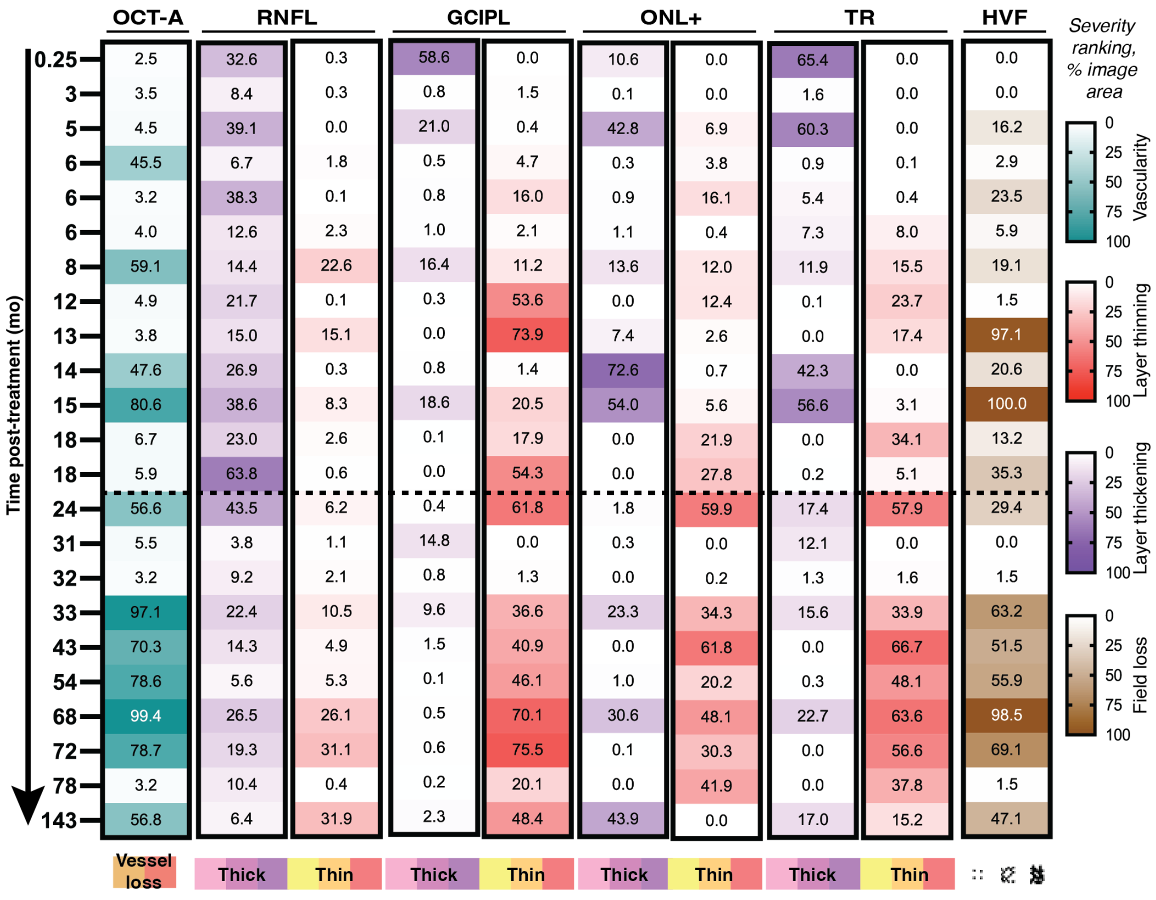

Supplementary Figure 3 (companion to manuscript Figure 4): Comparison of magnitude of pathology and dysfunction across outcome measures. The percentage of the pixels in the image area classified as abnormal by each severity ranking is listed in each cell and represented by a color scale (cyan, vessel density; red, layer thinning; purple, layer thinning; brown, visual field sensitivity). Larger numbers indicate more image area classified as abnormal. The dashed horizontal line demarcates the early (< 24 mo post-RT) and late (≥ 24 mo post-RT) cohorts, with data sorted chronologically in months from radiotherapy.

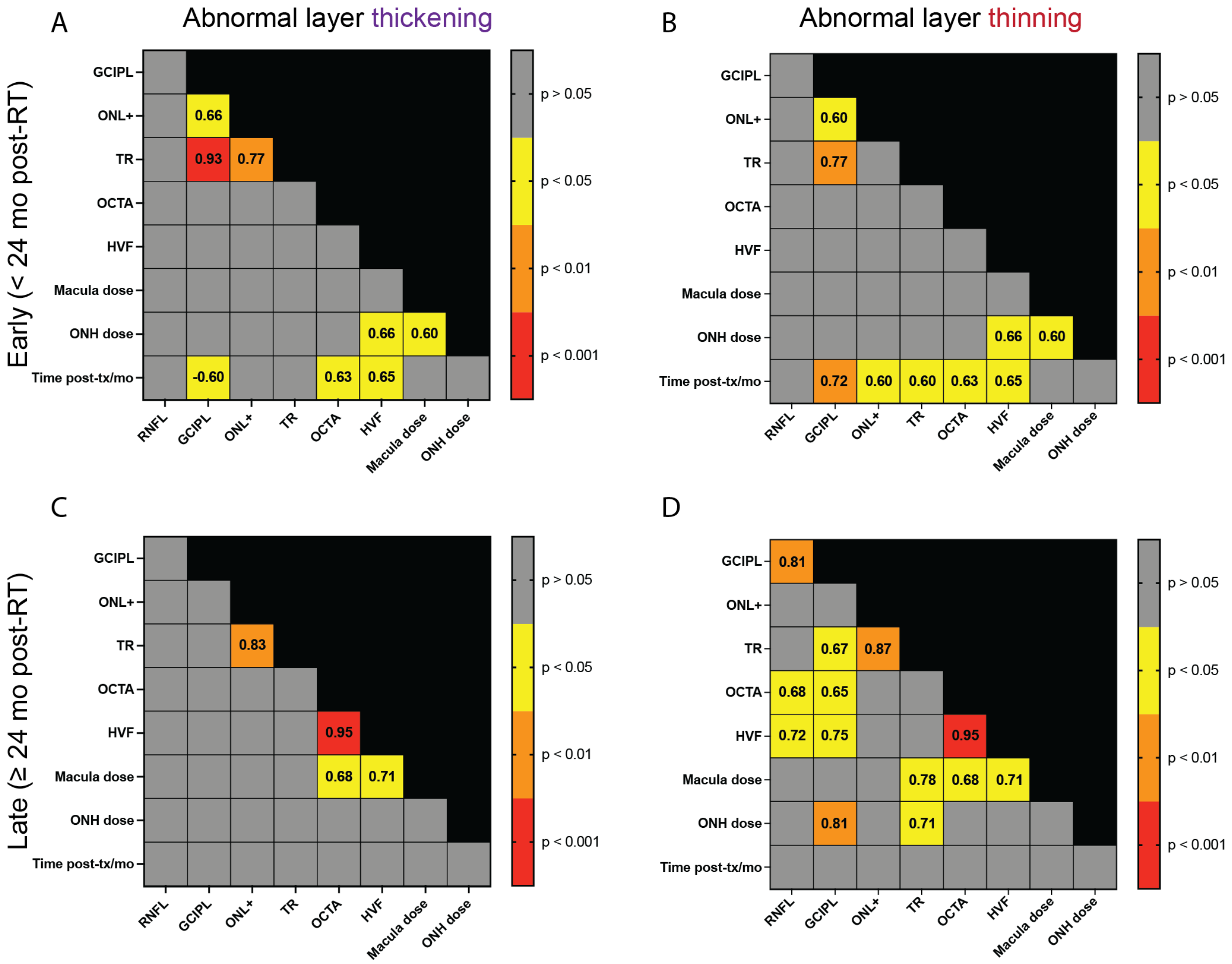

Supplementary Figure 4 (companion to manuscript Figure 5): Spearman correlation matrices of outcome measures separated by retinal layer pathology and time from radiotherapy. The percentage of imaged area occupied by abnormally thick (A, C) and abnormally thin (B, D) retina are compared to vessel density, visual field sensitivity, radiation dose to the macula and optic nerve regions, and time from radiotherapy. Correlations are further separated into early (A, B) and late (C, D) time groups. Each significant correlation ($p < 0.05$) is color-coded by p level and labeled with its Spearman r.
